# A Perinatal Review of Prevalence and Risk Factors for Substance Abuse Among Pregnant Women in Singapore

**DOI:** 10.7759/cureus.96085

**Published:** 2025-11-04

**Authors:** Ryan Wai Kheong Lee, Kai Wei Lee, Kok Hian Tan

**Affiliations:** 1 Obstetrics and Gynaecology, KK Women's and Children's Hospital, Singapore, SGP; 2 Obstetrics and Gynaecology, KK Women’s and Children’s Hospital, Singapore, SGP

**Keywords:** multidisciplinary approach, perinatal outcome, pregnancy, qualitative research, substance abuse

## Abstract

Introduction: Drug use during pregnancy can have detrimental effects on pregnancy and can lead to poor maternal and child health outcomes. There is currently limited information available on the prevalence and risk factors for drug abuse in pregnant women in Singapore. The aims of the study are to (i) establish the prevalence of drug abuse and (ii) assess risk factors for drug abuse during pregnancy.

Methods: A perinatal review of the management of cases of substance abuse in pregnancy from 2010 to 2020 was performed. Case records of substance abuse (excluding alcohol and tobacco) over the last 10 years from 2010 to 2020 were sourced via ICD-10 Diagnosis and extracted from the medical records. Essential demographic data were subsequently collected.

Results: There was a total of 20 women with perinatal drug abuse between 2010 and 2020. Three of the 20 women engaged in drug abuse for two of their pregnancies each, making 23 pregnancies affected by substance abuse during this period. The prevalence was low at 0.018% (23/128576) or 1 in 5,590 pregnant women. The common substance abuse was heroin with 11 cases (55.0%) and amphetamines/methamphetamines with nine cases (45.0%). High-risk factors for the pregnant substance abuser were younger age < 30 years old, Malay or Indian ethnicity and a history of smoking. In general, pregnant drug abusers book late in their pregnancy and tend to have little or no proper prenatal care and to default on their pregnancy follow-ups.

Conclusion: The prevalence of drug abuse among pregnant women in Singapore is low. The overall low prevalence of drug abuse nationally has contributed to low prevalence among pregnant women. Management of substance abuse in pregnancy with referrals to the appropriate multidisciplinary team for comprehensive assessment and individualized care in general have been satisfactory.

## Introduction

Substance abuse is defined as a pattern of harmful use of any substance and can be classified as mild, moderate and severe, depending on the number of criteria they fulfill, according to diagnostic criteria set out in the Diagnostic and Statistical Manual of Mental Disorders, fifth edition (DSM-V) [1]. Singapore has one of the lowest prevalence of substance abuse in the world with an estimated drug abuser prevalence of 83.7 per 100,000 population [2]. This is in part due to Singapore’s strict laws against drug abuse. However, the country remains vulnerable to the rise of the international drug trafficking industry. In 2018, the amount of methamphetamine (MA) seized increased to 228 ton-equivalents with worrying trends of increased trafficking in Southeast Asia. According to statistics provided by Central Narcotics Bureau (CNB) Singapore, the number of drug abusers decreased by 15% in 2020; however, the proportion of new drug abusers remained high at 38%, with 62% of them being under 30 years old. The most commonly abused drugs in Singapore in 2020 were MA, heroin, and new psychoactive substances (NPS) [3].

There are global concerns over increasing maternal opiate use and the role of substance use in pregnancy-associated deaths. In areas of high opioid use in the United States, such as Texas, up to 17% of pregnancy-associated deaths were attributed to substance abuse [4]. Consequently, the rising prevalence of opioid use in pregnancy has resulted in an increase in neonatal opioid withdrawal syndrome (NOWS) with 6.0 per 1,000 hospital births, costing an estimated $1.5 billion a year in healthcare costs [5].

Substance abuse among pregnant women imposes a significant socioeconomic burden, with increasing needs for medical and social services. Agarwal et al. reported an incidence of 0.25% (38 out of 14,690 births) of perinatal drug abuse in KK Women’s and Children’s Hospital, the largest public maternity hospital in Singapore, during the period of 1994-1996 [6]. This increasing global prevalence raises concerns for a possible increase in cases of pregnant substance abusers in Singapore.

As Singapore employs a holistic strategy with a harm-prevention approach for managing drug abuse, it is timely to examine the prevalence and management of drug abuse in pregnant mothers and their fetuses and/or newborns. To our knowledge, there has been no study done on perinatal drug abuse or substance abuse in pregnant women for nearly three decades in Singapore, since Agarwal et al. in 1996.

With a multi-disciplinary research team comprising obstetricians, neonatologists, pharmacists, and psychiatrists from our hospital, we performed a retrospective qualitative study on local cases of substance abuse in pregnancy at our hospital between 2010 and 2020. This study will enable us to gain insights into the management and counseling of pregnant women with substance abuse and to develop effective policies and guidelines to help this vulnerable population.

The aims of our study on our local population of patients were to establish the (i) prevalence of substance abuse in our hospital and by extension Singapore and (ii) risk factors for substance abuse during pregnancy.

## Materials and methods

A perinatal retrospective review on the management of substance abuse in pregnancy was performed from 2010 to 2020. All cases identified were sought for audit care review, conforming to Personal Data Protection Act (PDPA) regulation. All cases were subsequently de-identified for analysis. SingHealth Centralised Institutional Review Board (CIRB 2022/2001) approval was obtained for this study.

Data was obtained from our inpatient Datawarehouse computer system (SAP/IS-H/EHINTS). In this system, each patient’s details had been coded according to the International Classification of Diseases, 10th revision, Clinical Modification (ICD-10-CM) at discharge. Case records of substance abuse (excluding alcohol and tobacco) over 10 years from 2010 - 2020 were sourced via ICD-10 diagnosis codes. The following ICD-10-CM Diagnosis Code P96.1 - “Neonatal withdrawal symptoms from maternal use of drugs of addiction” and ICD-10-CM Diagnosis Code “O99.320 - Substance Drug use complicating pregnancy, unspecified trimester” were used.

The case records were obtained and essential demographic data collected included the following: age, race, gravida/parity, marital status, booking gestation, BMI, singleton/twins, previous history of live/stillbirths, smoking, alcohol, date of delivery, gestation at delivery, mode of delivery, baby weight, gender and APGAR scores. Additional data collected included the type of drug abuse, history of drug-taking before and during pregnancy, antenatal and/or perinatal complications from drug abuse including significant neonatal outcomes such as fetal anomalies, neonatal withdrawal syndrome and developmental issues. Individual case records were screened to look for general trends including protective and risk factors, socio-economic background and home situation.

Descriptive statistics were used to evaluate the distribution of demographic data. Comparisons of categorical variables were conducted using the chi-squared test, where appropriate.

## Results

There was a total of 20 women with perinatal drug abuse and they delivered a total of 37 babies (36 singleton pregnancies & one twin pregnancy) between 2010 and 2020.

Prevalence

Three (15.0%) of the 20 women engaged in substance abuse for two of their pregnancies each (whereas the other women denied repeated drug abuse in subsequent pregnancies). Thus, there were 23 pregnancies affected by drug abuse during this period, with one twin pregnancy, bringing the total number of involved babies to 24. There were 128,576 deliveries from 2010 to 2020. The prevalence of substance abuse during pregnancy was thus 0.018% (23/128576) or 1 in 5,590 pregnancies.

Notwithstanding the possibility of under-ascertainment and under-reporting, the prevalence was 13.9 times lower than a previous study of 0.25% (38/14690) in 1994-1996 in the same hospital [6], showing a significant decrease in prevalence (p-value < 0.001).

Substance abuse

The most commonly abused drug in pregnancy was heroin with 11 cases (55.0%), followed by nine cases of amphetamines/methamphetamines (MAs) (45.0%), three cases (15.0%) of buprenorphine (Subutex), two cases (10.0%) of sniffed glue and two cases (10.0%) of benzodiazepine abuse. Among the 20 women, three women (15.0%) were found to have repeated drug use in subsequent pregnancies and seven out of 20 women (35.0%) abused more than one substance simultaneously. Among the 11 women with heroin drug abuse, six mothers were reported to be on methadone therapy, and another one absconded before methadone therapy could be instituted.

Medical, obstetric and neonatal outcomes

Among the 20 pregnant women, there were two mothers who had Hepatitis C, but there were no records of any of the mothers having Hepatitis B, syphilis or HIV. There were concomitant medical issues with drug abuse in seven women with asthma, three with depressive disorders, two with bipolar disorder, one with personality disorder, and one with diabetic nephropathy.

One woman presented at 35 weeks gestation with heroin withdrawal but absconded and did not deliver in our hospital, and one woman presented at 28 weeks gestation for suspected benzodiazepine withdrawal but went back to her home country after discharge. Of the remaining 21 deliveries, the mean gestation at delivery was 37.1 weeks. Mode of delivery was 15 (71.4%) normal vaginal deliveries, 5 (23.8%) emergency cesarean sections (CS) and 1 (4.7%) elective CS.

Five of the 21 pregnancies (23.8%) suffered from obstetric complications: two premature preterm rupture of membranes, one poorly controlled diabetes in pregnancy, one placenta abruption, and one placenta accreta. A detailed breakdown of the 20 patients is seen in Table 1.

**Table 1:** Descriptive Summary of 20 Patients NVD: Normal vaginal delivery; LSCS: lower segment cesarean section

No	Number of Pregnancies	Age	Race/Ethnicity	Gestation	Mode of Delivery	Gravida/Parity	Type of drug used
1	1	26	Indian	37+4	NVD	G7P4	Inhalant use
2	2	26	Malay	40	NVD	G3P0	Heroin
3	1	21	Indian	39+3	NVD	G3P2	Alcohol, methamphetamine
4	1	33	Malay	34+4	Emergency LSCS for fetal distress	G10P3	IV Buprenorphine, heroin
5	2	37	Indian	32+0	Emergency LSCS for breech, intrauterine growth restriction, chronic placenta abruption	G5P3	Heroin
6	1	34	Chinese	39+1	NVD	G6P2	Buprenorphine, heroin, amphetamine
7	1	35	Chinese	38+2	Emergency LSCS for fetal distress	G3P2	Heroin
8	1	20	Malay	38+4	NVD	G2P1	Heroin
9	1	23	Malay	36+5	NVD	G5P2	Heroin
10	1	21	Malay	38+4	NVD	G4P1	Methamphetamine
11	2	22	Indian	40+1	NVD	G1P0	Heroin, methamphetamine, inhalant use
12	1	33	Indonesian	36+1	NVD	G5P2	Heroin, methadone
13	1	32	Chinese	38+0	NVD	G8P2	Methamphetamine
14	1	18	Malay	36+2	NVD	G1P0	Methamphetamine
15	1	38	Malay	37+2	NVD	G3P0	Heroin, methamphetamine
16	1	35	Indian	40+1	Emergency CS for scar pain and previous CS	G5P1	IV Buprenorphine, benzodiazepines
17	Twins	40	Chinese	34+2	Emergency CS for breech, twins, rupture of membranes	G2P0	Alprazolam and quetiapine – often overdose when stressed (especially Alprazolam)
18	1	18	Malay	40+1	NVD	G1P0	Methamphetamine
19	1	34	Malay		Elective CS for previous CS	G7P5	Heroin
20	1	35	Others (Sri Lanka)		Unknown	G3P1	Zolpidem

While there was no report of confirmed neonatal withdrawal syndrome in this group of 22 babies, there was one case of baby ‘jitteriness’ observed and a high proportion (50%) had related poor neonatal outcomes (e.g., prematurity, small for gestation, congenital anomalies, autism, neurodevelopmental delays and infant deaths). Nine were delivered prematurely, six had neurodevelopmental delay and/or autism, three had intrauterine growth restriction (SGA), and two passed away before six months of age. One baby also had congenital cytomegalovirus infection from maternal transmission.

Risk factors

The mean maternal age was 29.4 years (range 18 to 40 years, SD 7.3 years). This was a younger age mean compared to national maternal age averages in 2011 (29.9, 32.2, 34.0, 34.7 years for 1st to 4th order of birth). In terms of race, there was a higher proportion of Malays (9; 45.0%), followed by Indians (5; 25.0%), Chinese (4, 20.0%) and Others (2; 10.0%). In contrast, the average race proportions for the delivery population of the hospital during the time period were Malays 27.1%; Indians 11.2% and Chinese 41.6%, showing that Malays and Indians in this substance abuse population were much higher in proportion.

Among the 20 women, 13 (65.0%) were smokers and five (25.0%) consumed alcohol, of which one had alcohol intoxication during pregnancy.

## Discussion

Substance abuse during pregnancy often leads to adverse maternal and neonatal outcomes and the specific effects are dependent on the substance(s) of abuse. While the numbers remain relatively low in Singapore, substance abuse among women of childbearing age remains a concern. It is important to incorporate public and patient education to reduce the initiation of substance abuse and thereby mitigate the potential risks associated with pregnancies in this group. Globally, studies based on interviews or questionnaires (self-reported) showed a mean prevalence of 1.65% while studies based on toxicological analysis showed an average prevalence of 12.28% [7,8]. The prevalence of substance drug abuse among pregnant women in our study was 0.018% (23/128576) or 1 in 5,590 pregnant women, which was lower than similar global studies [7,8], and even lower than the already low rate in the only previous study in the same hospital [6].

Given the sensitive nature of substance abuse and given that it is illegal in Singapore, there is almost certainly underreporting of cases, as patients would be concerned about getting into trouble with the law. However, the clinical perception is that for the last decade, drug abuse in pregnancy has been rare. This low prevalence can be attributed to the strict laws and the local Central Narcotics Bureau’s strong efforts to reduce substance abuse, including good medical & preventive care and medical social worker support for pregnant women suffering from substance abuse.

From our study, we found that a large proportion of cases/patients have complex social issues, with a significant number being incarcerated and often defaulting on hospital follow-ups. Our case studies highlighted the many issues confronting the pregnant substance abuser - mental health struggles and difficulty coping with the care of their infant, who may be ill when born. Hence, it is paramount that clinicians work in close tandem with the medical social worker to provide strong support for this vulnerable group, to help care for them in their pregnancy and beyond. The medical social worker's role is critical, and they can help ensure follow-up of care, financial and legal issues for the substance user in pregnancy.

It is also paramount for healthcare providers to be made aware of the effects of these substances of abuse on mother and child, to allow early identification and initiation of harm prevention strategies by providing appropriate treatment and/or to counsel on cessation of the substance of abuse. Early intervention and initiation of appropriate support services may help to deter and minimize substance abuse during pregnancy.

It is pertinent to raise awareness of substance abuse in pregnant women and to use effective treatment regimens, such as methadone as for substitution therapy for opiates. There are maternal and fetal risks associated with drug withdrawal; hence, specialized programs for treatment and close medical monitoring are required. In addition, it is important to establish prevention strategies to minimize the incidence of drug relapse. This will require multi-disciplinary care, including medical social workers, psychologists, psychiatrists, and pharmacists, to coordinate care and therapy in these women. The neonatologists will need to observe closely for neonatal withdrawal syndrome after delivery. The development and update of guidelines for these relatively uncommon cases of substance abuse should be encouraged locally.

Substance abuse during pregnancy often leads to adverse maternal and neonatal outcomes [9-11]. Specific effects are dependent on the substance(s) of abuse. Drugs such as heroin, MA, cocaine, cannabis, 3,4-Methyl​enedioxy​methamphetamine (MDMA - also known as ecstasy), and ketamine have been on the rise and use during pregnancy have been shown to be associated with increased rates of syphilis [7,8] and other infections, which can lead to severe consequences such as stillbirth, neonatal death, fetal hydrops and congenital syphilis [12]. Our study showed that heroin and MAs were the most commonly used substances in pregnancy; hence we have focused on the effects of heroin and MAs in pregnancy in this current review.

Heroin

As heroin readily crosses the placenta, potential obstetric complications of prenatal exposure to heroin include spontaneous abortion, preterm labor, premature rupture of membranes, pre-eclampsia, placental abruption, intrauterine passage of meconium, intrauterine death, and toxaemia in heroin-dependent mothers [9-11].

Opioid use has also been linked to maternal death from drug overdose, primarily in the late post-partum period [13]. During the postpartum period, heroin-dependent mothers are at higher risk of relapse for drug use, whilst the higher prevalence of mental health conditions such as anxiety and mood disorders in opioid-dependent pregnant women also predisposes them to developing antenatal and postnatal depression [10,14].

There is uncertainty about whether maternal opioid use during pregnancy is teratogenic. In a systematic review of opioid use in pregnancy in 2017, 17 out of the 30 (case-control or cohort) studies demonstrated statistically significant positive associations between prenatal opioid exposure and at least 1 congenital malformation; the authors, however, had concerns over the quality of the studies [11]. There is little data specifically on fetal or neonatal outcomes on heroin alone (rather than opioids as a class), as addicted mothers often exhibit polysubstance drug abuse. In addition, heroin is often adulterated with various substances, thus it is challenging to discern the effects of heroin specifically.

The most commonly reported fetal outcome with heroin use is NOWS, previously known as neonatal abstinence syndrome [8,15,16]. Exposure to heroin in utero causes hyperactivity of the central and autonomic nervous system, and a sudden withdrawal after delivery causes symptoms including irritability, high-pitched cries, tremors, hypertonia, sleep disturbances, poor feeding, gastrointestinal disturbances, sweating, yawning, and hyperthermia [17]. This usually occurs within the first 28 days of life and sometimes may require further treatment. In opioid-dependent users, NOWS may affect up to 45-94% of newborns and results in a 7-fold increase in neonatal intensive care stays [8,15,16,18]. Fortunately, in our population of 11 maternal heroin abusers, there were no confirmed cases of NOWS, although one baby had some “jitteriness” and was observed for a few days in the special care nursery.

Heroin use has been associated with intrauterine growth retardation, infections, respiratory distress syndrome and lower Apgar scores, premature birth, reduced head circumference, and increased sudden infant death syndrome [18-21]. Hulse et al. observed an increase in relative risks in neonatal mortality with illicit heroin use; although this is likely associated with the high-risk lifestyle of heroin abuse, rather than the use of heroin itself [20].

Methamphetamines and amphetamines

MA is commonly known as ‘ICE’ by local drug abusers and can come in crystallized or tablet form. It can be taken in various ways, including inhalation, injection, orally, and rectally. The drug is a stimulant that is highly addictive and leads to euphoria and disinhibition, and long-term use may cause anxiety, depression, weight loss, dental issues, and even psychosis [22]. Women taking MAs may be more prone to high-risk behavior due to disinhibition, leading to higher rates of unwanted pregnancies and sexually transmitted infections [22].

The Infant Development, Environment, and Lifestyle (IDEAL) study is the largest prospective, longitudinal study to date with 412 mother-infant pairs at four sites that examined maternal, infant, and long-term behavioral outcomes of maternal MA use. There were no reported differences in obstetric complications such as fetal distress, chronic hypertension, pre-eclampsia or prolonged rupture of membranes [23], but MA use is significantly associated with small for gestation age babies [22,23]. Women using MA are also much more prone to both antenatal and postnatal depression, which may affect the neurodevelopment of their infants [24]. The babies are less likely to be breastfed and may demonstrate decreased arousal, increased stress, and poorer suckling ability [22,23,25].

Though MA use has not been clearly linked to teratogenicity [22], there are concerns about the potential impact on long-term neurodevelopment of the exposed children. Prenatal exposure to MA has been linked to reduced height growth velocity and delayed gross motor development between 0-3 years old [26,27], increased externalizing behavioral issues and risk factors for ADHD at five years old and up to 7.5 years [28-30]. However, at 7.5 years old, early adversity rather than prenatal MA exposure alone was associated with behavioral problems, likely due to the challenging social circumstances in which these children grew up [30].

Although the prevalence of pregnant substance abusers in this study was fortunately small and thus difficult to elucidate more statistically significant factors, there were some typical patterns within this pregnant drug abuser population. In our study, 10% (2/20) of women had given birth to four or more children and had children with different partners. These women were more likely to have booked late in their pregnancies, defaulted on their pregnancy follow-ups, or been admitted to hospital multiple times, but without attending subsequent outpatient follow-ups; hence, these pregnant drug abusers tended to have little to no proper prenatal care.

Risk factors for drug abuse

Possible predisposing factors for drug abuse for this group were psychiatric issues, including depressive disorders, bipolar and personality disorders, and a history of suffering physical abuse. They often also suffered from marital/relationship issues, poor social support, and poor finances. Substance abuse was often used as a means to cope with their multiple stressors, which were compounded by the physical and emotional stress of pregnancy, making it very difficult for them to stop their substance abuse even if they wished to.

It is difficult to identify whether poor maternal and fetal outcomes are directly related to drug use, or rather the combination of factors that define many substance abusers, such as limited prenatal care, polysubstance use, multiple incarcerations, poor socio-economic background, poor social support, and relationships, all of which lead to a non-conducive environment for both pregnancy and bringing up their children. Risk factors such as age and ethnicity cannot be taken in isolation as they are intimately linked to socio-economic factors such as low income and lower educational attainment; for example, based on the 2020 Population Census, 27.7% of the Malay population aged 25 years and above had diploma or degree qualifications, compared to 49.9% of the Chinese population and 56.3% of the Indian population [31], although this is almost double the proportion (15.3%) of the 2010 Malay population.

Given the potential for long-term effects on mother and child, substance abuse among women of childbearing age remains a concern despite the relatively low numbers in Singapore. A key message from this qualitative study is to recognize that the management of perinatal substance abuse is often complicated and requires a close multi-disciplinary collaboration. This includes obstetricians, psychiatrists and psychologists, pediatricians and neonatologists, physicians, allied health professionals, addiction counselors, and nurses.

The medical social worker's role is critical, and they can help ensure follow-up of childcare, financial, and legal issues and provide strong support in this vulnerable group to help care for them in their pregnancy and beyond. The management of substance abuse in pregnancy is complicated given its close relationship with the law - hence, working closely with the prison medical service is also important for patients who are in custody.

Tackling this issue effectively will need to be a multi-pronged approach, incorporating patient care, education, research and policies which include the following: (1) Enhancing public and patient education to reduce the initiation of substance abuse and thereby mitigate the potential risks associated with unplanned pregnancies in this group, (2) Creating pathways for early identification of substance abuse, incorporating increased awareness among healthcare providers, early screening tools, and protocols for toxicology screening (universal vs symptom-driven vs at-risk groups), (3) Performing further research and audits of the effects of drugs specifically on Singapore/Asian cohorts, as majority of the published research and systematic reviews are based on Caucasian populations, (4) Increasing clinical training on use of medication for opioid use treatment and safe administration, and (5) Developing specially designed programs and workflows to provide in-house multi-disciplinary care once pregnant substance abusers are identified. Early intervention and initiation of appropriate support services may help to deter and minimize substance abuse during pregnancy.

Limitations

The number of cases identified from 2010 to 2020 is almost certainly an underestimate, with cases not being picked up, as ICD code diagnosis is provider-dependent, hence likely to be missed in the medical records. With the small numbers, this gives us an idea of trends and potential risk factors, but it cannot be definitively used to draw conclusions.

As the study is a retrospective review of case records, many of which may be incomplete, we were mostly able to look at non-modifiable risk factors. Future prospective studies may be able to incorporate more validated screening tools and social and psychological factors.

Additionally, many guidelines, including those of the American College of Obstetricians and Gynecologists (ACOG), recommend early and universal screening, and there are screening instruments that can be used for such purposes [32,33], such as CAGE for alcohol dependence and CRAFFT for adolescent alcohol and cannabis abuse. However, substance abuse (whether pregnant or not) is illegal and harshly punished in Singapore; thus, pregnant users are unlikely to disclose any possible substance abuse unless they are caught by law enforcement, further decreasing the number of cases detected.

## Conclusions

Substance abuse in pregnancy in our hospital is rare, with a low prevalence of 0.018% between 2010 and 2020. The most common drug abused was heroin, followed by MAs. Risk factors for substance abuse identified in Singapore include younger age < 30 years old, Malay or Indian ethnicity, a history of smoking, and concomitant psychiatric disorders. Establishing national guidance on the identification and management of substance use disorders in pregnancy remains an important priority. Enhancing awareness, providing comprehensive multi-disciplinary care and early intervention ensure access to preventive and treatment services to optimize clinical outcomes for pregnant mothers with substance abuse.
